# Probiotic *Bacillus amyloliquefaciens* C-1 Improves Growth Performance, Stimulates GH/IGF-1, and Regulates the Gut Microbiota of Growth-Retarded Beef Calves

**DOI:** 10.3389/fmicb.2018.02006

**Published:** 2018-08-28

**Authors:** Renjia Du, Shengyin Jiao, Yue Dai, Jianbo An, Jia Lv, Xiaoni Yan, Juan Wang, Bei Han

**Affiliations:** ^1^School of Public Health, Health Science Center, Xi’an Jiaotong University, Xi’an, China; ^2^Institute of Food and Agriculture, China National Institute of Standardization, Beijing, China; ^3^Xi’an Center for Disease Control and Prevention, Xi’an, China

**Keywords:** growth-retardation, *Bacillus amyloliquefaciens*, *Bacillus subtilis*, intestinal microbiota, endocrine hormones

## Abstract

Growth retardation of calves is defined as a symptom of impaired growth and development, probably due to growth hormone disorder as well as natural and environmental factors in livestock. The growth-promoting effects of probiotics were determined in 50 growth-retarded growth calves. They were supplied with *Bacillus amyloliquefaciens* C-1 (Ba, 4 × 10^10^CFU/d, *n* = 16), *B. subtilis* (Bs, 4 × 10^10^CFU/d, *n* = 18), and negative control (NC, *n* = 16) for 30 days. Pre- and post-intervention, the growth performance (weight gain rate, feed intake and feed conversion rate) was analyzed, the serum GH, IGH-1 and immunoglobulin levels were assayed, and the fecal microbiota was detected. Calves in Ba and Bs groups demonstrated increased body weight gain, feed intake and GH/IGF-1 levels, as well as a more efficient feed conversion rate, compared with NC group (*P* < 0.05). Additionally, the abundances of bacteria contributing to the production of energy and SCFAs (short chain fatty acids), including *Proteobacteria, Rhodospirillaceae*, *Campylobacterales*, and *Butyricimonas* were increased compared with NC group (*P* < 0.05, *FDR* < 0.1); and the suspected pathogens, which included *Anaeroplasma* and *Acholeplasma* were decreased (*P* < 0.05, *FDR* < 0.1) in both the Bs and Ba groups. *Akkermansia*, which is involved in the intestinal mucosal immune response, was increased in Bs group after intervention (*P* < 0.05, *FDR* < 0.1), but exhibited no obvious difference in Ba group. The increased bacterial genera in Ba group were *Sphaerochaeta* and *Treponema* (*P* < 0.05, *FDR* < 0.1). These results indicate that the probiotics *B. amyloliquefaciens* and *B. subtilis* exhibited similar therapeutic potential in terms of growth performance by regulating hormones, and improving the intestinal and rumen development in growth-retarded animals.

## Introduction

Hybrid Qinchuan cattle and Japanese black cattle are intensively cultivated to supply the beef market in northwest China (Jiao et al., 2017). However, the refractory growth retardation occurs in beef cattle with normal birth weights, and causes great economic loss. Young animals with growth retardation are associated with extremely low feed conversion efficiency, high morbidity and mortality, which lead to increased farming costs and reduced production profit (Hu et al., 2016). A variety of studies have been performed with growth-retarded calves and have implicated somatotropic axis hormone secretion deficiency (Hu et al., 2016), as well as environmental factors, including food deprivation, malnutrition and disease (Fawzi et al., 1997; Hoffman, 1997; Mourits et al., 1997).

Animal growth is primarily regulated by GHRH, SS, GH, IGFs and related receptor and downstream pathways (Renaville et al., 2002). There are significant correlations between GH/IGF-1 and increased weight based on data reported from humans, pigs, and yaks (Owens et al., 1999). Direct injection of GHRP-1 improves endogenous growth retardation in yaks (Hu et al., 2016). Sugar supplementation was proved to stimulate the growth performance in growth-retarded Japanese black calves (Sato et al., 2010). However, some of the results have been rather difficult to apply directly to farm conditions, and do not completely reverse growth retardation.

The gut microbiota plays important roles in digesting food and absorbing nutrients from the host diet, regulating host fat storage, stimulating intestinal epithelial renewal and directing immune system maturation (Zhang et al., 2013), and it is reported recently that the synthesis of IGF was modulated by the host microbiota-derived metabolites (short chain fatty acids) to influence growth (Yan and Charles, 2018). Antibiotics used in animal breeding introduce problems while improving growth performance (Simoneit et al., 2014). The administration of antibiotics whether therapeutically or prophylactically, disturbs the normal microbiota balance of the host, which may cause growth retardation of the animal (Lupindu et al., 2015). Probiotics are used as a safe alternative to antibiotics to rebalance the intestinal flora and are supplemented in the diet to prevent diseases, improve inflammation and digestion and promote growth (Marques-Lopes et al., 2001; Riggs et al., 2006; Ford et al., 2014; Kalil and Schooneveld, 2014). The mechanisms underlying these beneficial effects on the host have been verified and involved interference with potential pathogens, improvement of barrier function, immunomodulation and production of neurotransmitters (Sánchez et al., 2016). Currently, lactic acid bacteria and *Bacillus subtilis* are used as feed additives to improve growth performance and immune function in some animals. However, controversy remains regarding the preparation safety and impact on young animals, particularly in terms of the strains, dosage and duration of probiotic administration, which should be carefully considered (Ritchie and Romanuk, 2012). Identifying effective probiotic bacteria as feed additives and untangling how these probiotics affect the host intestinal microbiota and immunity to improve health and performance are essential steps for the successful application of probiotics in calf production (Li et al., 2017).

*Bacillus amyloliquefaciens* is a particularly promising feed supplement, and it does not require strict growth conditions which including temperature, humidity, oxygen, fermentation medium, and is easy to be processed, very stable at host gastrointestinal tract (Hong et al., 2005). It produces several extracellular enzymes to augment the digestibility and absorption of nutrients in addition to overall intestinal immune function (Gould et al., 1975; Gracia et al., 2003; Guerra et al., 2007). In most cases, it has been used to protect plants against the bacterial and fungal pathogens in both soil and hydroponic applications (Molohon et al., 2011), and may prevent infection through competitive exclusion or by out-competing the unwanted pathogens (Tan et al., 2016). However, limited studies have demonstrated the oral administration of *B. amyloliquefaciens* fermentations as a feed additive during cattle production, and there have been no reports in growth-retarded cattles (Schofield et al., 2018). Thus, it remains unclear whether the probiotics-induced alternative flora microbiota improves growth retardation, and the corresponding mechanisms are unknown. To identify potential candidate probiotics for growth-retarded calves, this study evaluated the probiotic effect and potential mechanisms during dietary supplementation with *B. amyloliquefaciens* C-1 in growth-retarded calves as a practical assessment, and the corresponding effects were also compared with the commercial product of *B. subtilis*.

## Materials and Methods

### Bacterial Strains and Culture Conditions

*Bacillus amyloliquefaciens* strain C-1 (16S rRNA accession no. JX028840 in GenBank) was isolated from ready-to-eat sliced apple samples by the Food Microbiology Lab of the Nutrition and Food Safety Engineering Research Center of Shaanxi Province, Xi’an, China (the China Center for Type Culture Collection, CCTCCM2012177). A single colony of C-1 was picked and inoculated into LB medium at 30°C for 12 h, and sub-cultured in fermentation medium (12.4 g/L tryptone, 20 g/L glucose, 5 g/L NaCl, 1.5 g/L K_2_HPO_4_, 0.04 g/L MnSO_4_, 1.7 g/L FeSO_4_, and 1.2 g/LMgCl_2_, pH7.2–7.4) for 72 h. The fermentation culture was then prepared as a vacuum freeze-dried powder. *B. subtilis* commercial product (4.5 × 10^9^ CFU/g, Q/YB.J02-62-2014) was obtained from Fubon^®^ (Angel Yeast Co., Ltd., China).

### Design of an Intervention Experiment in Growth-Retarded Calves

The study was conducted from April to June in 2016 at a scaled bovine livestock farm with more than 10,000 calves on hand. The farm is located on 108°0′52.70″ east longitude and N34°18′40.75″ north latitude in Yangling, Shaanxi Province, China. The cattle strain assessed was a crossbreed of Japanese black cattle and Red Angus cattle (male parent), and Qinchuan cows (female parent). All calves were inspected upon receipt to ensure no deformity or early disease signs. Only the animals aged 3–6 months with body weights less than 1.5 times of the reference body weight of calves at the same age were used. The farm owner gave consent for the animals to be used in this study.

Fifty calves aged 3–6 months with growth retardation were collected, and randomly divided into three groups: the negative control group (*n* = 16, normal diet, NG), *B. subtilis* feeding group (*n* = 18, Bs, 4 × 10^10^CFU/d + normal diet), and *B. amyloliquefaciens* C-1 feeding group (*n* = 16, Ba, 4 × 10^10^CFU/d + normal diet). *B. subtilis* and *B. amyloliquefaciens* C-1 were mixed separately into the supplied fodder. All the calves were supplied with same regular diet in the same barn; the different intervention treatments continued for 30 days.

Body weight was measured prior to morning feeding every week (days 0, 7, 15, 22, and 30). Calves feed five times per day according to daily feeding standards, and daily feed intake was recorded. The ADG was calculated with following formula:

$$ADG=\frac{\left(\text{Final weight-Initial weight}\right)}{\text{days}}$$

Before and after intervention, blood samples and fecal samples were collected to blood biochemistry, immunological parameters and gut microbiota, separately. Blood samples were collected from the jugular vein prior to the morning feeding, and serum was isolated from the blood after centrifuging at 3,000 rpm and then stored at -20°C for further analysis. Fecal samples were collected from all calves by rectal massage, and then placed on ice and transported back to lab within 4 h.

This experiment was approved by Animal Care and Use Committee of Xi’an Jiaotong University (No. 2015-227). All procedures and the use of animals were carried out in accordance with the Guide for the Care and Use of Laboratory Animals prepared by the Institutional Animal Care and Use Committee of Xi’an Jiaotong University, Xi’an, China.

### Blood Serum Immunological and Endocrine Hormone Parameters

IgA, IgG, Ig-M, IGF-I, and GH concentrations in the collected blood serum samples were measured by using enzyme-linked immunosorbent assay kits specific to calve (Nanjing Jiancheng Technology Co., Ltd., Nanjing, China) with an automatic microplate reader (TECAN Infinite^®^ 200, Switzerland). Each sample was assayed in triplicate with matrixing through the standard curve.

### Analysis of the Gut Microbiota

Microbial genomic DNA was extracted from 220 mg of each fecal sample using a QIAamp Fast DNA Stool Mini Kit according to the manufacturer’s recommendation. Successful DNA isolation was confirmed by 1% agarose gel electrophoresis. The 16S V3–V4 region was amplified using the primers U341F (3′ACT CCT ACG GGA GGC AGC AG5′) and U806R (3′GGA CTA CHV GGG TWT CTA AT5′). PCR amplification was carried out in 50 μl reaction mixtures containing 0.5 U of ExTag Hotstart DNA polymerase (TaKaRa Inc., Dalian, China), dNTPs at 50 μM, each primer at 25 μM, 5 μl of premix buffer (containing 20 mM MgCl_2_), and 50 ng of DNA as template. PCR was performed on an automated thermocycler (Bio-Rad MyCycler, United States) for 30 cycles consisting of 30 s at 94°C, 30 s at 50°C, and 30 s at 72°C; Amplicons were visualized on 1.5% agarose and checked for length; products with the desired size (approximately 460 bp) were purified using a QIAquick gel extraction kit (QIAGEN, Germany). DNA quality and amounts were assessed using an Invitrogen Qubit^®^ dsDNA BR kit. The experiments were performed in triplicate. The purified fragments in each sample were normalized and pooled. The 16S rDNA high-throughput sequencing was performed by the Realbio Genomics Institute (Shanghai, China) using the Illumina HiSeq platform. The sequenced data were deposited into the Sequence Read Archive (SRA) of NCBI^1^ and can be accessed via accession number SRP127590.

The raw data were then subjected to a quality control procedure using UPARSE. The qualified reads were clustered to generate OTUs at the 97% similarity level using USEARCH. A representative sequence for each OTU was assigned to a taxonomic level in the Ribosomal Database Project (RDP) by the RDP classifier. Principal component analysis and heatmap analysis were performed using R3.1.0. Linear discriminant analysis coupled with effect size (LEfSe) was performed to identify the bacterial taxa differentially represented between groups at the genus level or higher taxonomy levels. Profiling of the predictive gut microbiota was analyzed by using PICRUSt.

### Statistical Analysis

Statistical analyses were performed using the *t*-tests and analysis of variance (ANOVA) with JMP pro (SAS Institute Inc., NC, United States), STAMP^10^ and SPSS V20.0 (IBM Inc., IL, United States). Data of growth performance are presented as the means ± SEM. Differences were considered significant at *P* < 0.05.

The Wilcoxon rank-sum test was used to analyze the significant different taxa between different groups. Wilcox.test was used between two groups analysis, and kruskal.test was used among three groups analysis. In the multiple testing, *P-*value correction via the Benjamini–Hochberg false discovery rate (FDR) was performed and FDR < 0.1 was considered significant.

## Results

### Contribution of Probiotics to the Growth of Growth-Retarded Calve

**FIGURE 1:**
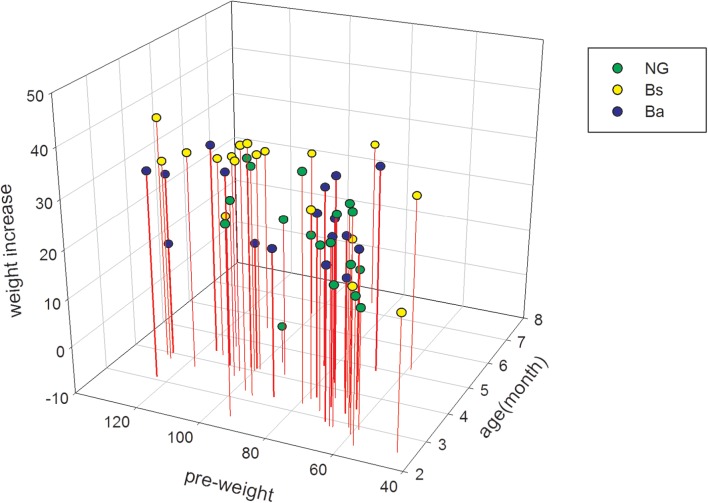
The age specific weight increasing among different groups pre- and post-intervention.

The initial body weight among NG, Bs, and Ba group showed no obvious difference (*P* = 0.06), the average age had difference (*P* = 0.04), then take the consideration of body weight and age together using SPSS, there had no obvious difference among three groups (*P* = 0.06). After intervention for 30 days, the body weights and feed intake greatly improved (**Table 1**). The ADG values for the Bs, Ba, and NG groups were 0.99, 0.85, and 0.53, respectively. The increasing in age-specific weight was analyzed among groups pre- and post-intervention (**Figure 1**). Compared with the NG group, the changes in growth development-related indicators fluctuated significantly in the Bs and Ba groups. The corresponding feed conversion rate decreased obviously (Bs 2.47, Ba 2.80, and NC 3.54), the Bs intervention contributed the most to feed conversion (*P* < 0.05).

**Table 1:** Effects of *Bacillus* spp. on performance in calves with growth retardation.

	NG	Bs	Ba	*P*-value
	(*n* = 16)	(*n* = 18)	(*n* = 16)	
Initial BW (kg)	78.25 ± 3.76	95 ± 5.62	90.75 ± 5.77	0.06
Final BW (kg)	94.31 ± 4.78	124.83 ± 6.77	116.37 ± 6.09	0.03
BW gain (Kg)	16.06 ± 1.61	29.83 ± 1.66	25.63 ± 1.57	0.04
ADG	0.53 ± 0.05	0.99 ± 0.06	0.85 ± 0.05	0.03
Feed intake (kg)	56.89 ± 2.38	73.54 ± 1.89	71.83 ± 1.83	0.02
Feed conversion rate	3.54 ± 0.12	2.58 ± 0.13	2.97 ± 0.22	0.04


### Serum Immunological Parameters and Growth Performance

**FIGURE 2:**
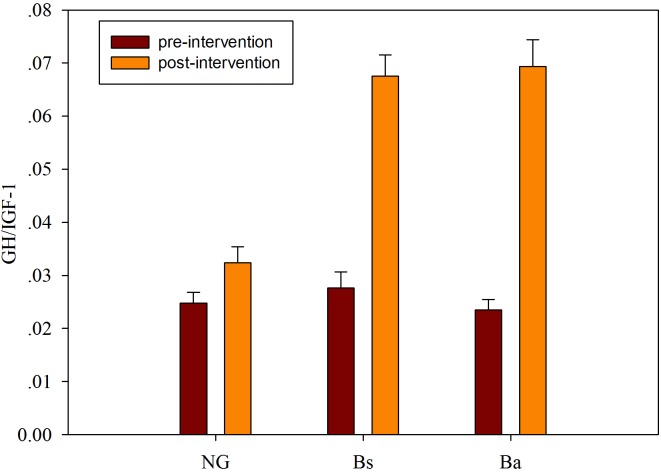
The GH/IGF-I level changes after intervention among different groups.

In calves supplemented with *B. amyloliquefaciens*/*B. subtilis*, the serum IgA, IgM, and IgG levels were increased compared with the NG group, although not significantly (*P* > 0.05). Additionally, growth factors were detected before and after probiotics administration for 30 days, IGF-1 and GH were decreased among all three groups, while GH/IGF-1 levels increased after intervention. There was a significant correlation between serum GH/IGF-1 and calf body weight, and there were differences in GH/IGF-1 among the intervention groups (*P* < 0.05); the contribution of the Ba intervention was higher than that of Bs (**Figure 2**).

### Gut Microbiota DNA Sequence Data and Quality Control

A total of 4,832,522 paired-end 250-bp reads were acquired. Pre- and post-intervention, there were 778,424 and 771,612 clean reads in NG calves; 854,253 and 860,688 clean reads in Bs calves; 780,261and 787,284 clean reads in Ba calves, respectively. Based on the collected quality-controlled clean reads, a total of 1,899 OTUs were identified from all calf fecal samples with 97% species similarity. Pre- and post-intervention, there were 1141 and 1180 OTUs in NG calves; 1200 and 1288 OTUs in Bs calves; 1159 and 1218 OTUs in Ba calves, respectively. Most OTUs were shared among groups at the same age, only 7, 25 and 16 OTUs were uniquely identified in calves from the NG, Bs and Ba groups pre-intervention, while 40, 37, and 17 OTUs were uniquely identified in calves from the NG, Bs and Ba groups post-intervention, respectively (**Table 2**).

**Table 2:** Results of OTU, species richness and diversity of microorganism communities.

	Intervention					
	
	NG group		Bs group		Ba group	
			
	Pre-	Post-	Pre-	Post-	Pre-	Post-
Clean reads	778,424	771,612	854,253	860,688	780,261	787,284
OTUs	1141	1180	1200	1288	1159	1218
Chao1	435.43	479.33	528.82	552.19	510.57	527.50
Observed_species	327.44	386.88	417.44	433.28	412.69	415.31
PD_whole_tree	25.57	27.44	29.21	30.32	29.56	29.38
Shannon	5.5	6.39	6.6	6.64	6.43	6.51
Simpson	0.87	0.96	0.97	0.97	0.96	0.97
Goods coverage	0.99	0.99	0.99	0.99	0.99	0.99


### The Gut Microbiota Pre-intervention

**FIGURE 3:**
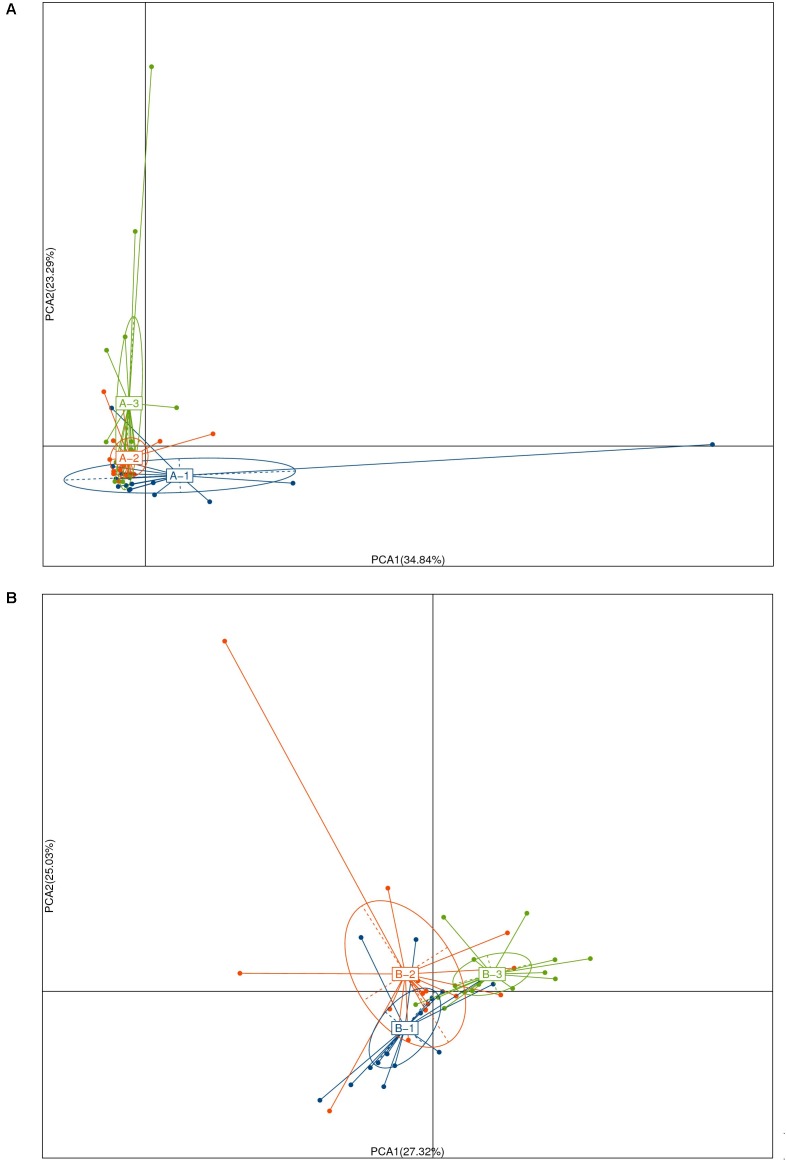
Principal coordinate analysis of unweighted Unifrac community distances for intestinal microbiota of the pre- **(A)** and post- **(B)** intervention among NG(1), Bs(2), and Bs(3) groups.

The sequenced data (NG, *n* = 16, 1141 OTUs; Bs, *n* = 18, 1200 OTUs; Ba, *n* = 16, 1159 OTUs) were analyzed. First, the distribution of age and sex in each group was uniform, and the distribution of OTUs was significantly different pre- and post-intervention among three groups (**Figure 3**). The cattle were analyzed based on the similar age (L, <3.5 months; H, ≥3.5 months) among the groups. The microbiota OTUs in calves younger than 3.5 months changed greatly, while the OTUs were relatively stable in calves more than 3.5 months old (**Supplementary Figure S1**). Among each group, there were 1124 uniform OTUs (59.9%). Prior to intervention, the most dominant phyla in the three groups of calves were similar, consisting of Bacteroidetes, Firmicutes, and Proteobacteria, and more than 80% classes were Clostridia and Bacteroidia, while more than 40% of genera consisted of *Bacteroides*, *Escherichia/Shigella*, *Paraprevotella*, and *Clostridium*.

### Shift in Community Membership After *Bacillus* Intervention

**FIGURE 4:**
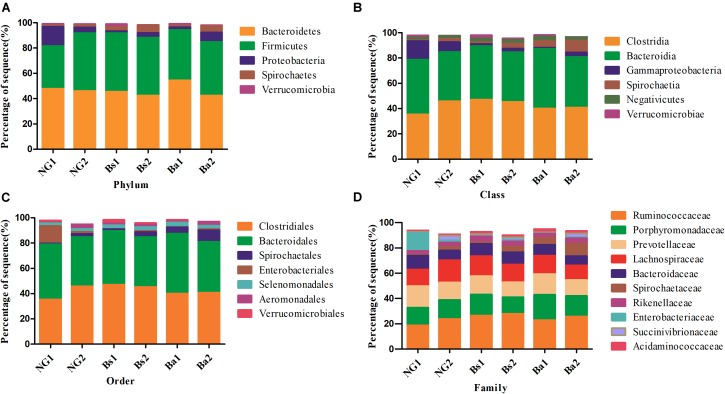
Bacteria distributions using V3–V4 amplicon sequencing at phyla **(A)**, class **(B)**, order **(C)**, family level **(D)**; 1-pre-intervention, 2-post-intervention.

After intervention for 30 days, a total of 20 phyla were shared by calves from all groups (**Supplementary Figure S1**). Firmicutes was the most dominant the phylum (*P* < 0.05) in all samples, followed by Bacteroidetes and Proteobacteria. In the NG group, Fibrobacteres, Cyanobacteria/Chloroplast, Acidobacteria, Lentisphaerae, Deferribacteres, Nitrospirae, Lentisphaerae, and Deinococcus-Thermus were appeared after intervention. In the Bs group, Thaumarchaeota, Cyanobacteria/Chloroplast, Acidobacteria and Gemmatimonadetes were uniquely arised after intervention; and in the Ba group, Fusobacteria was identified after intervention. Synergistetes was identified in both Bs and Ba groups. The bacterial abundance of Firmicutes in calves of the NG group increased from 35.28 to 45.52% after intervention (*P* = 0.025, *FDR* = 0.032), whereas Synergistetes decreased to 0.01% (*P* = 0.048, *FDR* = 0.055). In the Bs group, Actinobacteria was significantly decreased (*p* = 0.002, *FDR* = 0.066). In the Ba group, Spirochaetes (*P* = 0.004, *FDR* = 0.029) and Elusimicrobia (*P* = 0.047, *FDR* = 0.082) were increased, whereas Bacteroidetes (*P* = 0.001, *FDR* = 0.018) was decreased from 47.57 to 42.86%. Additionally the bacterial abundance of Spirochaetes in calves from the Ba group (7.84%) was higher than that of NG (2.24%) and Bs (3.51%) groups, whereas Tenericutes was lower (**Figure 4A**). Proteobacteria has a lower abundance in the Ba and Bs groups compared with NG group (*P* = 0.044).

After 30 days of intervention, the most dominant classes (relevant abundance >1%) in the Bs group are Clostridia, Bacteroidia, Negativivutes, Spirochaetia, Gammaproteobacteria, Verrucomicrobiae, and Bacilli. The bacterial abundances of Gammaproteobacteria and Spirochaeta were significantly increased from 1.76 to 2.94% (*P* = 0.006, *FDR* = 0.082), and 1.29 to 3.90% (*P* = 0.003, *FDR* = 0.066), respectively. While in the Ba group, the most dominant classes (relevant abundance >1%) were Bacteroidia, Clostridia, Spirochaeta, Negativicutes and Gammaproteobacteria, while Gammaproteobacteria and Methanobacteria were significantly increased (**Figure 4B**).

At the order level, Clostridiales, Bacteroidales, Spirochaetales, Selenomonadales, Verrucomicrobiales, Aeromonadales, and Bacillales were the most dominant (relevant abundance >1%) in the Bs group, whereas Spirochaetales significantly increased from 1.29 to 3.93% (*P* = 0.007, *FDR* = 0.061). In the Ba group, the most dominant classes (relevant abundance >1%) were Clostridiales, Bacteroidales, Spirochaetales, Selenomonadales, and Aeromonadales. In calves in the NG group, the most dominant classes (relevant abundance >1%) were Clostridiales, Bacteroidales, Aeromonadales, Pseudomonadales, Spirochaetales, Selenomonadales and Enterobacteriales, whereas Aeromonadales was significantly increased (**Figure 4C**).

The most dominant families (relevant abundance > 1%) in the Bs group were Ruminococcaceae, Lachnospiraceae, Porphyromonadaceae, Prevotellaceae, Bacteroidaceae, Spirochaetaceae, Rikenellaceae, Succinivibrionaceae, Acidaminococcaceae, Veillonellaceae, Clostridiaceae, Planococcaceae, Verrucomicrobiaceae, and Enterobacteriaceae. Spirochaetaceae significantly increased from 1.40 to 4.28% (*P* < 0.01). In the Ba group, the most dominant families (relevant abundance >1%) were Ruminococcaceae, Porphyromonadaceae, Prevotellaceae, Lachnospiraceae, Spirochaetaceae, Bacteroidaceae, Rikenellaceae, Succinivibrionaceae, Acidaminococcaceae, Clostridiaceae, and Methanobacteriaceae. Clostridiaceae and Methanobacteriaceae were significantly increased from 0.16 to 1.52% (*P* = 0.001, *FDR* = 0.020) and 0.25–1.12%, respectively (*P* = 0.003, *FDR* = 0.063) (**Figure 4D**).

At the genus level, the 20 most abundant genera were *Bacteroides, Escherichia/Shigella*, *Paraprevotella*, *Prevotella*, *Clostridium XlVa*, *Alistipes*, *Alloprevotella, Succinivibrio*, *Roseburia*, *Treponema*, *Acinetobacter*, *Clostridium sensu stricto*,*Oscillibacter, Phascolarctobacterium, Barnesiella, Akkermansia, Parabacteroides, Anaerovibrio, Clostridium XlVb*, and *Ruminococcus.* The bacterial abundances of *Succinivibrio* (*P* = 0.031, *FDR* = 0.023), *Roseburia* (*P* = 0.004, *FDR* = 0.018) and *Ruminococcus2* (*P* = 0.036, *FDR* = 0.061) in calves of the NG group were obviously increased after 30 days, whereas *Clostridium XlVb* was decreased (*P* = 0.022, *FDR* = 0.061). In calves of the Bs group, *Alistipes* was increased (*P* = 0.035, *FDR* = 0.076), and *Paraprevotella* (*P* = 0.039, *FDR* = 0.083), *Phascolarctobacterium* (*P* = 0.012, *FDR* = 0.066) and *Akkermansia* (*P* = 0.015, *FDR* = 0.045) were decreased. In calves of the Ba group, *Akkermansia* (*P* = 0.000, *FDR* = 0.005) and *Clostridium XlVb* (*P* = 0.015, *FDR* = 0.006) were obviously increased, whereas *Clostridium XlVa* was decreased (*P* = 0.001, *FDR* = 0.039).

For the microbiota changed obviously, we had them analyzed further based on the similar age (L, <3.5 months; H, ≥ 3.5 months) among the groups. Among the NG-L, Bs-L, and Ba-L groups, NG-H, Bs-H, and Ba-H groups, there had no difference in the average age (*P* = 0.6716, *P* = 0.1073) (**Supplementary Figure S1**). After 30 days, except the above description, the intervention of *Bacillus* spp. greatly increased Clostridia, Spirochaetia, and Verrucomicrobiae, and decreased Gammaproteobacteria of calves in NG-L group (**Supplementary Figure S2**).

### Changes in the Microbiota Among Groups After Intervention

When the gut microbiota structure was compared among the three groups post-intervention, *Spirochaetes* was the only genus that increased, and *Tenericutes* was the only genus that decreased in the Ba group. *Lentisphaerae*, *Deferribacteres*, and *Nirospirae* were only present in the NG group and were not detected in the Bs and Ba groups. *Synergistetes* was only present in the Bs and Ba groups and was not detected in the NG group. The bacterial abundance of *Treponema* in calves of the Ba group was significantly higher than that in the NG and Bs groups (*P* = 0.002, *FDR* = 0.074). *Roseburia* was increased in the Ba and Bs groups compared with the NG group (*P* = 0.021, *FDR* = 0.092).

Comparison of the gut microbiota structure of the three groups post-intervention showed that the bacterial abundances of *Akkermansia*, *Butyricimonas* and *Pseudoflavonifractor* were higher in the Bs group, and *Spirochaeta* and *Treponema* were higher in the Ba group, while the abundances of *Acholeplasma* and *Anaeroplasma* were lower in the Bs group. Between the Bs and Ba groups, the abundances of *Akkermansia* and *Pseudoflavonifractor* were higher in the Bs group, while *Spirochaeta* and *Treponema* were higher in the Ba group. There were no significant differences in alpha diversity based on an analysis of the Shannon and Chao1 indexes. Taking into account the sequence abundances of all samples as determined by a non-parametric test, the most significant difference between groups arose from the Ba intervention.

### Predicted Functional Metagenomes Analysis

**FIGURE 5:**
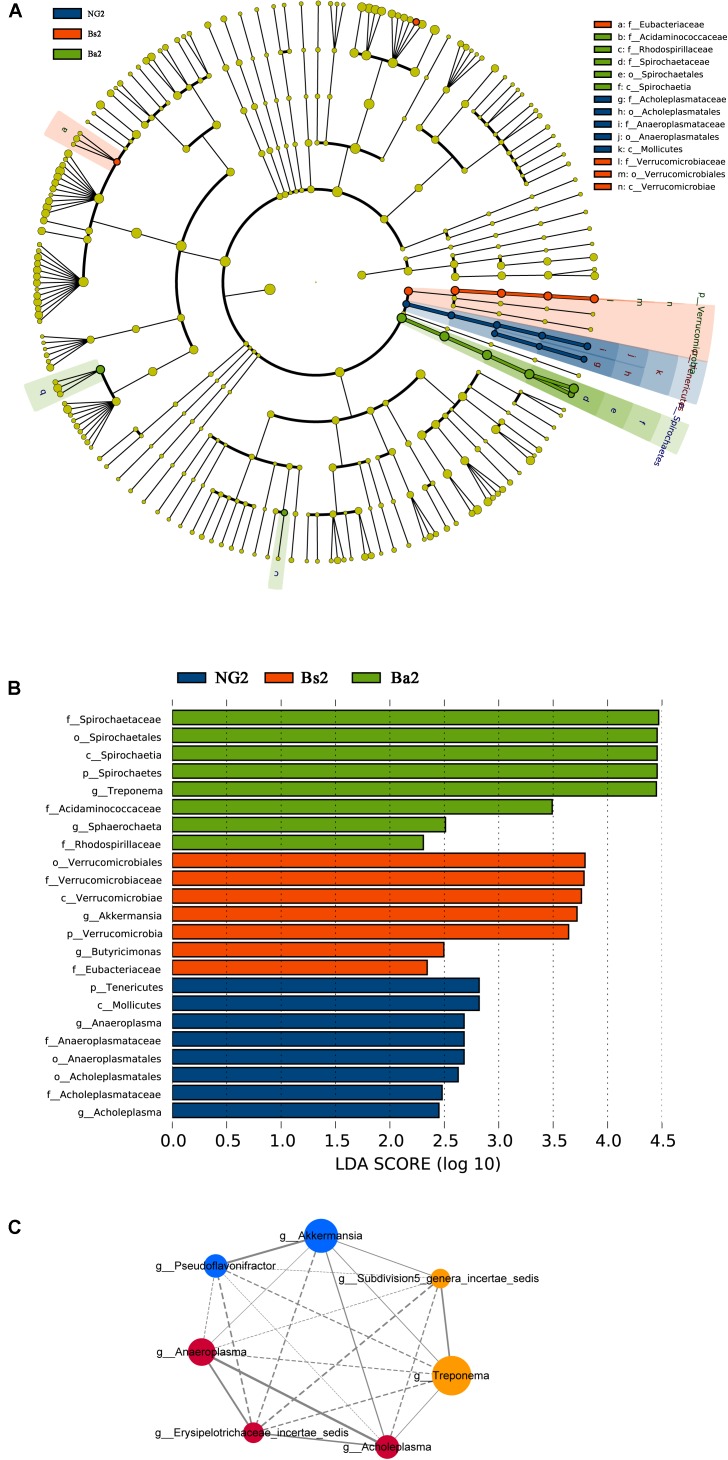
Bacterial taxa significantly differentiated **(A,B)** and Network analysis applied to the calf gut microbiota **(C)** among NG, Bs, and Ba groups post-intervention, identified by linear discriminant analysis coupled with effect size (LEfSe); for the network, node size is proportional to the genera abundance, node color corresponds to genera taxonomic classification (RED for NG, BLUE for Bs, and YELLOW for Ba group). Active line represents positive correlation and dotted line means negative correlation.

**FIGURE 6:**
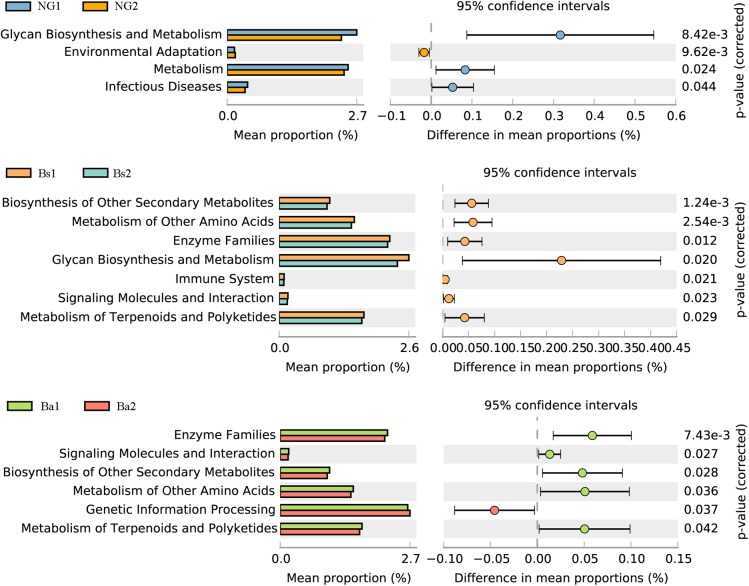
Functional genes of the intestinal microbiome which showed significant differences (*P* < 0.01) between NG(A), Bs(B), and Ba(C) groups at the phyla level.

To identify bacterial taxa that were significantly different between groups, LEfSe was performed. Among the abundant genera, *Clostridium XlVa* was abundant before intervention, and *Treponema*, *Caryophanon*, and *Clostridium sensu strict* were enriched after intervention in the Bs group (**Figure 5A**); in the Ba group, *Clostridium XlVa* and *Akkermansia* were enriched before intervention, and *Treponema*, *Clostridium sensu stricto*, and *Methanobrevibacter* increased after intervention (**Figure 5B**). Network analysis indicated that *Anaeroplasma*, *Erysipelotrichaceae incertae sedis* and *Acholeplasma* have a strong positive relationship, and *Akkermansia*, *Pseudoflavonifractor*, and *Treponema* have a strong positive relationship (**Figure 5C** and **Supplementary Figure S3**). To assess the metabolic potential of the fecal microbiome, OTUs were entered into PICRUSt, and the inferred gene families were annotated against KOs and then collapsed into KEGG pathways. After *Bacillus* spp. intervention, the most abundant metabolic pathways were primarily associated with the biosynthesis of other secondary metabolites, the metabolism of other amino acids, enzyme families, signaling molecules and interactions, and the metabolism of terpenoids and polyketides, both in the Bs and Ba groups (**Figure 6** and **Supplementary Figure S3**).

## Discussion

Although they have no major clinical problems, some calves receive a low price at market because of retarded growth, and most calves with retarded growth are associated with extremely low feeding conversation efficiency, higher morbidity and mortality, which lead to increased breeding costs. In general, growth-retarded calves may suffer from saccharometabolic failure (Sato et al., 2010). It is well known that the administration of probiotics regulates and influences the diversity, composition and relative abundance of the intestinal flora, resulting in increased immune capacity, reduced incidence of digestive diseases, and improved nutrient digestion and absorption. Previous studies revealed that daily oral supplementation with *Lactobacillus fermentum* I5007 for neonatal piglets reduced the incidence of diarrhea, improved growth performance, favored intestinal development and altered the intestinal microbiota (Liu et al., 2014). And those effects were verified mostly in the healthy not the growth-retarded animals. In this study, the oral administration of *B. amyloliquefaciens*/*B. subtilis* improved the production performance, including increased GH/IGF-1 levels and bacterial diversity, of growth-retarded calves after intervention for 30 days (**Figure 2**).

Serum immunoglobulins, including IgA, IgG, and IgM, are produced by B-lymphocytes, which are the major impact factors of humoral immunity, to prevent and resist infection. Serum immunoglobulin content is an important indicator of humoral immunity. The gastrointestinal tract supplies the largest number of immune cells; therefore, any factors that modify the gut situation will influence immunity (Altmeyer et al., 2014). It was reported that supplementation with *L. casei* and *Enterococcus faecalis* in sucking piglets increased serum IgA and IgG, but no treatment effects were observed for IgM (Liu et al., 2017). In our results, the function of growth-retarded calves greatly improved, and GH/IGF-1 was increased after intervention, but serum immunoglobulins demonstrated no obvious changes (*P* > 0.05). Growth retardation is associated with disorders of GH secretion and the host immune response, and recovery from these disorders requires a considerable amount of time (more than 30 days). The growth hormone GH/IGF-1improved more rapidly under probiotics intervention than did immunoglobulin levels in growth-retarded calves.

Previous studies have reported that the diversity, composition and relative abundance of the intestinal flora are influenced by probiotic administration. Feeding probiotics to animals may regulate microbial diversity, which is often associated with gastrointestinal disorders and rumen development during the early growth period (Sato et al., 2010; He et al., 2015). In the present study, feeding a *B. amyloliquefaciens*/*B. subtilis* diet to growth-retarded calves aged 3–6 months for 30 days increased the bacterial diversity compared with the negative control group, and structure tends to be more stable than that of the young cattle in NG group. And the adjustment of microbiota by probiotics seems more sensitive and effective at the growth-retarded cattle more than 3 months.

As shown by fecal bacterial sequencing results, most OTUs were shared between groups, indicating that OTUs that were unique to each group were more likely to be less abundant, but their functions are very important. Bacterial richness and diversity were increased compared with the NG group, and the overall microbial community structure (β-diversity indices) shifted after intervention (*P* < 0.05). Bacterial richness and diversity in the Bs group appeared to be lower than that of the Ba group on day 30, probably because *B. amyloliquefaciens* C-1produceslipopeptides to inhibit pathogens and modulate other components of the gut microbiota (Yang et al., 2015).

Bacteroidetes, Firmicutes, and Proteobacteria were the most dominant phyla in this study. Here, we showed that C-1 administration had an effect on the proportions of dominant phyla. The bacterial abundances of Spirochaetes and Euryarchaeota in calves from the Bs and Ba groups were both higher than the abundances in NG calves after intervention. It was previously reported that strict anaerobes (*Bacteroides* spp., *Clostridium* spp., and *Bifidobacterium* spp.) are the major intestinal bacteria in calves, generally reaching levels 100-fold higher than facultative anaerobes (Enterobacteriaceae) (Hanning and Diaz-Sanchez, 2015). As we showed in this study, the predominant genera in each of the samples were strict anaerobes, such as *Clostridium*, *Bacteroides*, *Ruminococcus*, *Alistipes*, *Prevotella*, and *Akkermansia*. In agreement with this study, a previous report showed that *Bacteroides*, *Oscillibacter*, *Alistipes*, *Ruminococcus*, and *Clostridium* were the predominant bacteria in the guts of buffalo and calves (Zhang et al., 2017). The gastrointestinal tracts of herbivores contain various microbes that harbor a complex lignocellulosic degradation system for microbial attachment and digestion of the plant biomass (He et al., 2015). For example, *Bacteroides* spp. digests a wide variety of otherwise indigestible dietary plant polysaccharides (Xu et al., 2003). In addition, *Alistipes* may be of interest in the field of fiber degradation (Peng et al., 2015). *Ruminococcus* are also considered to be the most important cellulose-degrading bacteria in the intestines of herbivores, and they produce large amounts of cellulolytic enzymes, including exoglucanases, endoglucanases, glucosidases, and hemicellulases (Singh et al., 2014). Accordingly, the enhanced detection of *Alistipes*, *Bacteroides*, and *Ruminococcus* in this study of the intestinal microbiota in beef calves is associated with fiber degradation in feed. Normal bovine rumen fluid contains relatively large numbers of spirochetes capable of fermenting polymers that are commonly present in plant materials. After intervention, among the three groups, the Ba group saw a significant increasing in the phylum Spirochaetes and the genus *Treponema* (4.43% in the NG group, 8.12% in the Bs group, and 17.2% in the Ba group) (*P* < 0.01). It was previously reported that bacteria affiliated with *Treponema* were negatively correlated with methane emission and with the improvement in animal production traits in Holstein cows (positive correlations with digestible dry matter intake and digestible organic matter intake).

After intervention, *Succinivibrio* and *Butyricimonas* increased in the Bs and Ba groups. In the rumens of cattle fed high starch-containing diets, *Succinivibrio dextrinosolvens* usually improves ruminal starch digestion. This bacterium also produces succinate during rumen fermentation, and this succinate is decarboxylated by *Selenomonas ruminantium* to form propionate, a major ruminal SCFA (Hespell, 1992). In addition to acting as local substrates for energy production, SCFAs have diverse regulatory functions. *Butyricimonas* produces butyric acid, which is an important energy source not only for the gut microbiota itself but also for IECs. Propionate has been shown to be a signaling molecule that improves epithelial development in calves (Zhang et al., 2018). With the exception of SCFAs, nutrient absorption is related to the function of the host intestinal mucosa. After intervention, *Akkermansia* spp. content was more notably increased in the Bs group compared with the NG group. *Akkermansia* spp., a next-generation beneficial microbe, play an important role in mucus degradation and production, and has potential anti-inflammatory properties in host intestines (Belzer and de Vos, 2012; Cani and de Vos, 2017). Additionally, the enrichment of *Akkermansia* in the Bs group may indicate the contributing effects of intestinal mucosal immunity and nutrient absorption of growth-retarded calves.

Therefore, we developed a hypothesis suggesting that feeding probiotics (*B. subtilis* and *B. amyloliquefaciens*) to growth-retarded calves increases the rate of fiber degradation, which in turn stimulates the flow of the digesta, the impetus to eat and GH secretion, ultimately improving growth performance. There were some limitations in this study; for example, the intervention time was not sufficient to show the immunoglobulin response or real changes in the ruminal microbiota. Future surveillance studies will prioritize these issues. And more growth-retarded animals will be incorporated in the next study with longer observation time to verify the results.

## Conclusion

The results of this intervention experiment showed that probiotics increased the feed intake of growth-retarded calves, enhanced their body weights with a higher GH/IGF-1 ratio and increased the number of intestinal fiber-degrading bacteria. In growth-retarded calves with no birth defects (immune system defects and/or genetic abnormalities), supplementation with probiotics (especially *Bacillus* spp.) contributes to the stabilization of intestinal microbiota, improves growth performance and increases the farming output value, especially for the young animals more than 3 months.

## Availability of Data and Material

The sequenced data were deposited into the Sequence Read Archive (SRA) of NCBI (https://www.ncbi.nlm.nih.gov/sra) and can be accessed via accession number of SRP127590.

## Author Contributions

All authors contributed to data interpretation, wrote and revised various parts of the paper. RD, SJ, JL, XY, and JW conducted the work presented here and performed data analysis. YD and BH drafted the manuscript. YD, JA, and BH revised the overall paper. BH supervised the work. All authors read and approved the final manuscript.

## Conflict of Interest Statement

The authors declare that the research was conducted in the absence of any commercial or financial relationships that could be construed as a potential conflict of interest.

## Footnotes

1 https://www.ncbi.nlm.nih.gov/sra

## Supplementary Material

FIGURE S1 The age distribution analysis of L (<3.5 months) and H (≥3.5 months) among three intervention groups **(A)**. There is no difference in NG-L, Bs-L, and Ba-L (*P* = 0.6716), and no difference in NG-H, Bs-H, and Ba-H (*P* = 0.1073). The PCoA analysis of the OTU difference among three groups after intervention in L **(B)** and H **(C)** separately. blue, NG group, red, Bs group, green, Ba group.

FIGURE S2 Bacteria distributions using V3–V4 amplicon sequencing at phyla **(A,B)**, class **(C,D)**, order **(E,F)**, family level **(G,H)**; L-Pre, lower age before intervention; L-Post, lower age after intervention; H-Pre, higher age before intervention; H-Post, higher age after intervention.

FIGURE S3 The correlation of the enriched microbiota changed pre- and post-intervention with metabolic pathways by R corrplot. **(A)** NG group, **(B)** Bs group, and **(C)** Ba group.

## Abbreviations

ADG: average daily gain
GH: growth hormone
GHRH: growth hormone releasing hormone
IECs: intestinal epithelial cells
IGF-I: insulin-like grows factor I
IGFs: insulin-like grows factor
OTU: operational taxonomic unit
PCoA: principal coordinates analysis
SCFAs: short-chain fatty acids
SS: somatostatin
