# Supplementary figures and images for: Probiotic Bacillus amyloliquefaciens C-1 Improves Growth Performance, Stimulates GH/IGF-1, and Regulates the Gut Microbiota of Growth-Retarded Beef Calves
Source: Front Microbiol. 2018 Aug 28;9:2006. doi: 10.3389/fmicb.2018.02006 (PMC6120984; doi:10.3389/fmicb.2018.02006)

Fig.S1

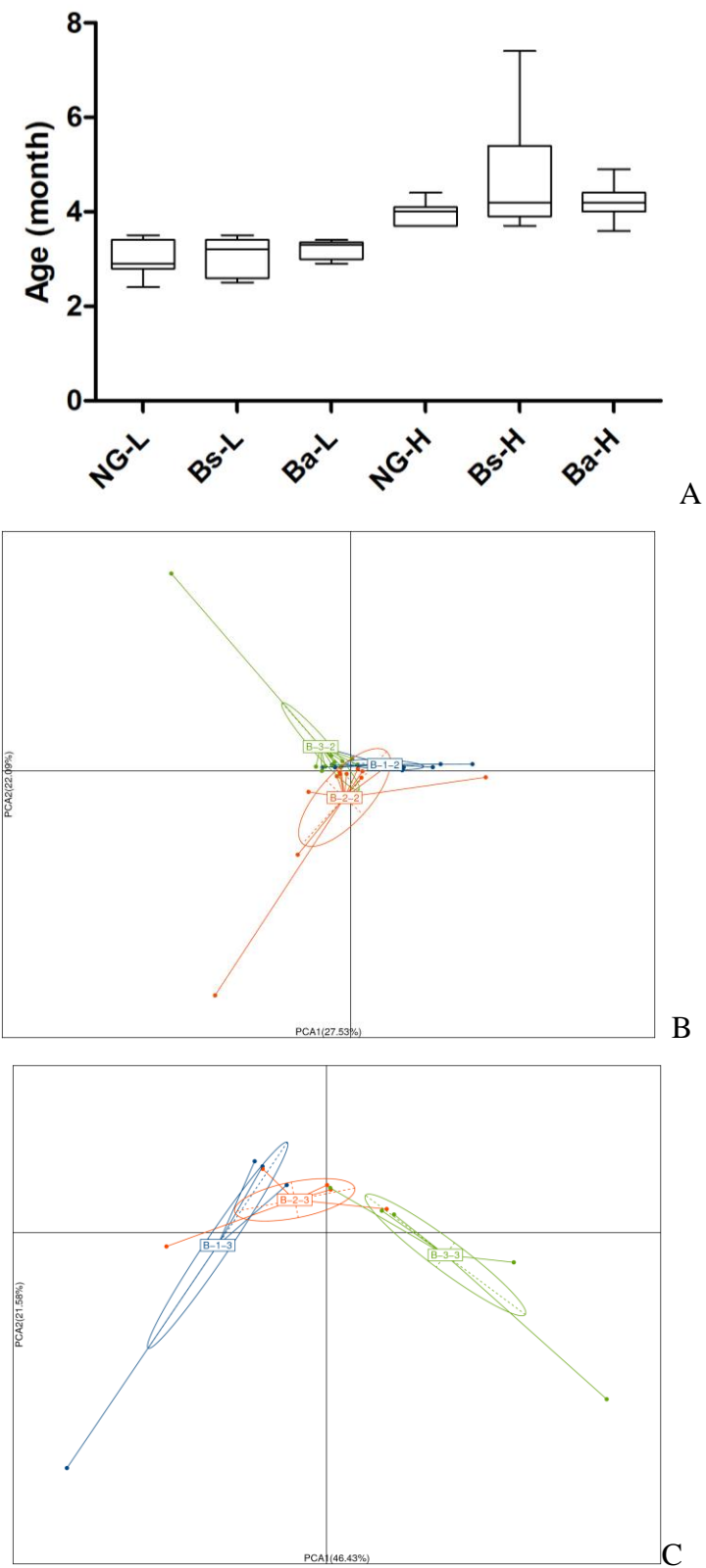

Fig.S2

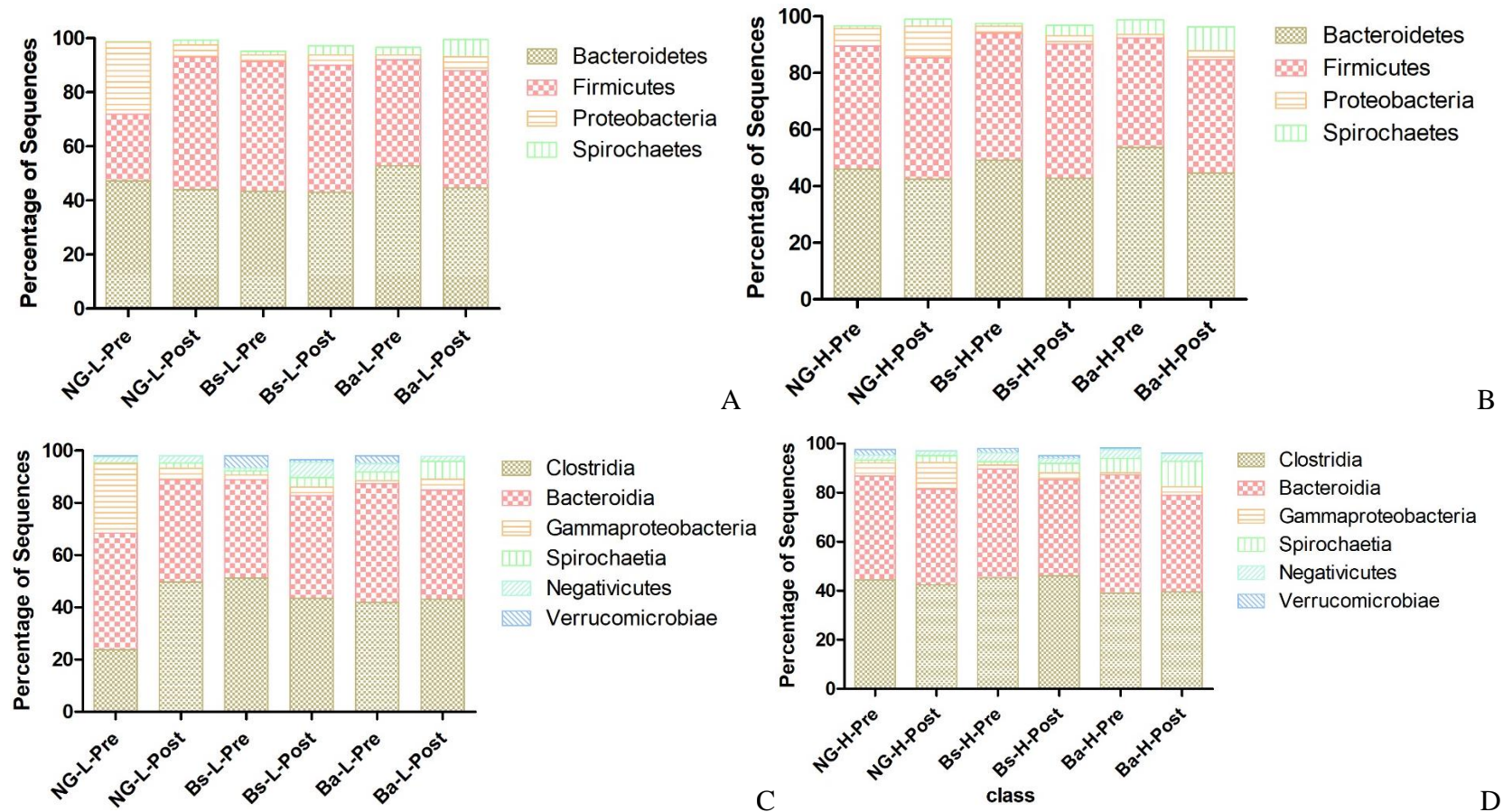

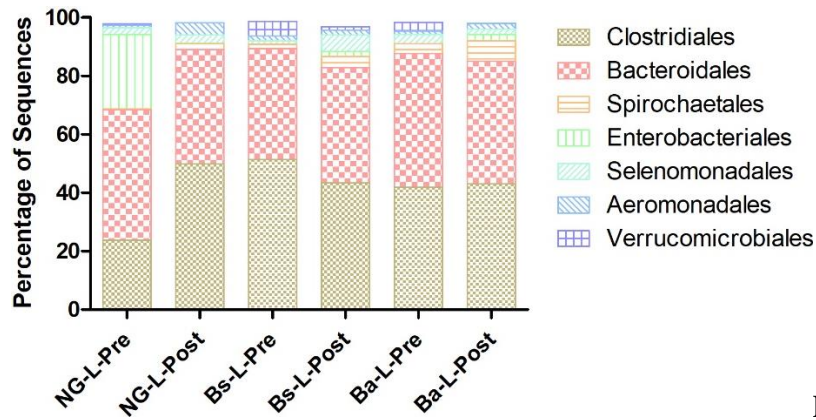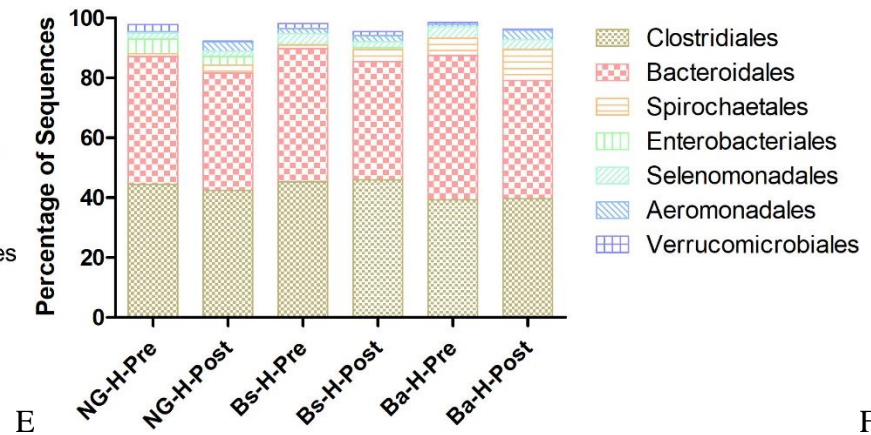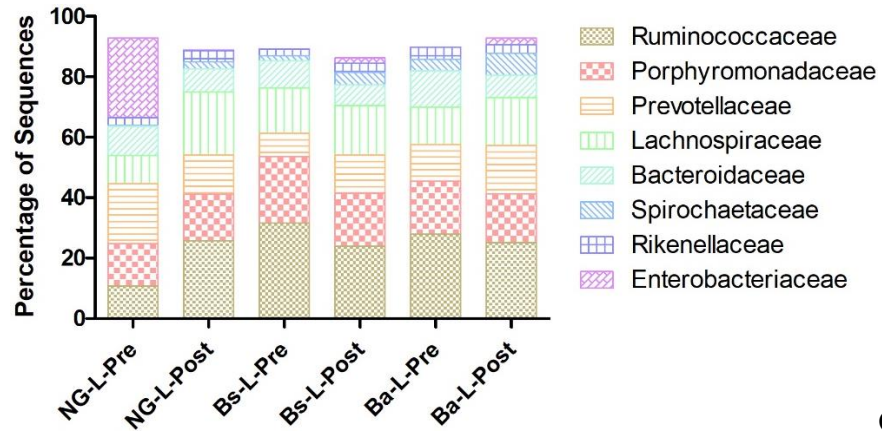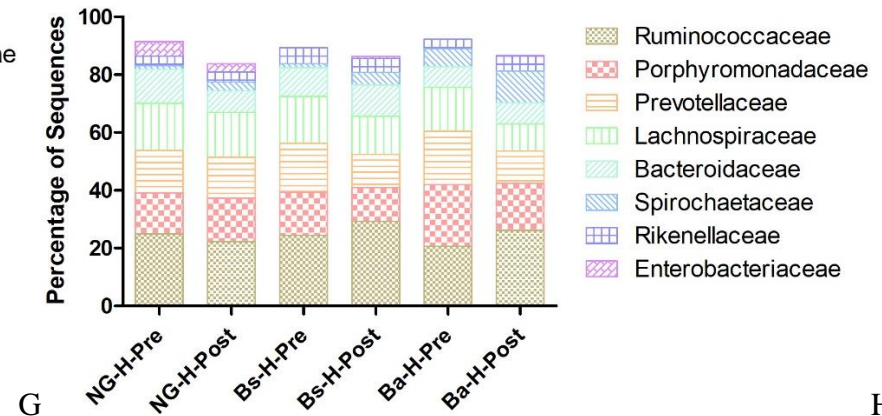

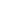 Pre-  
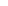 Post-

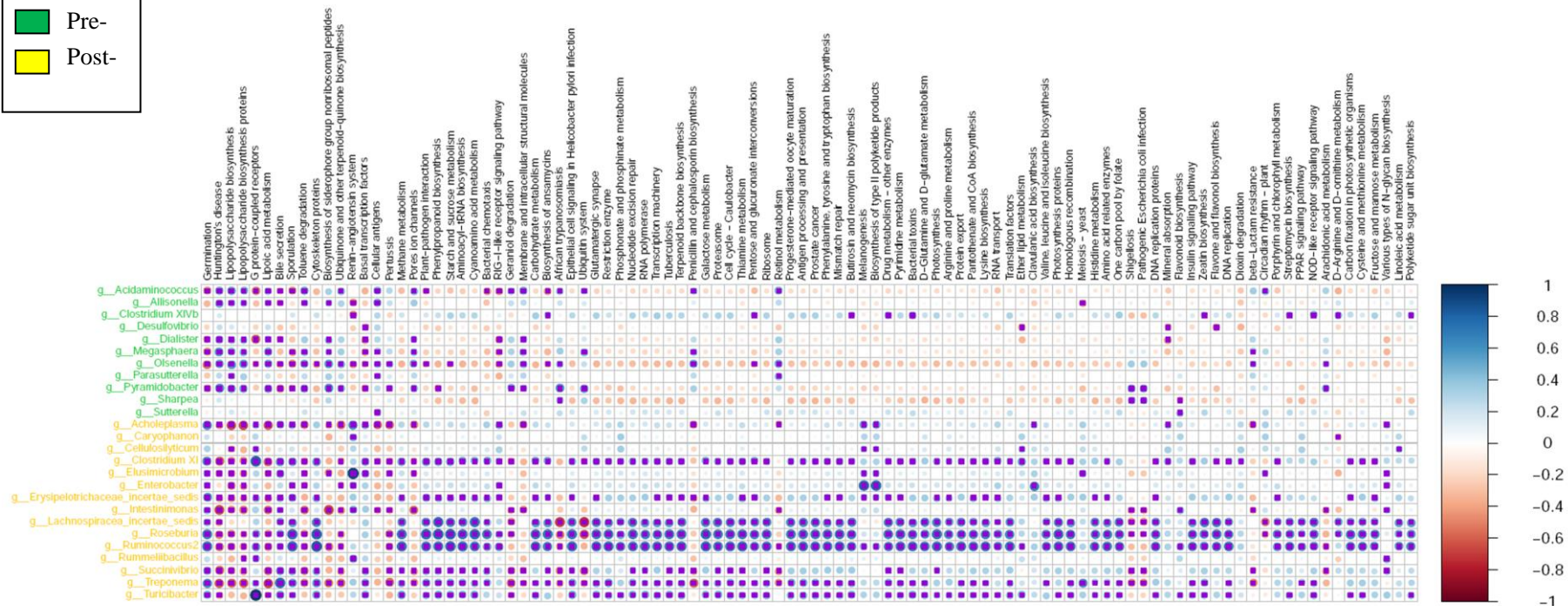

A

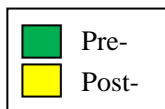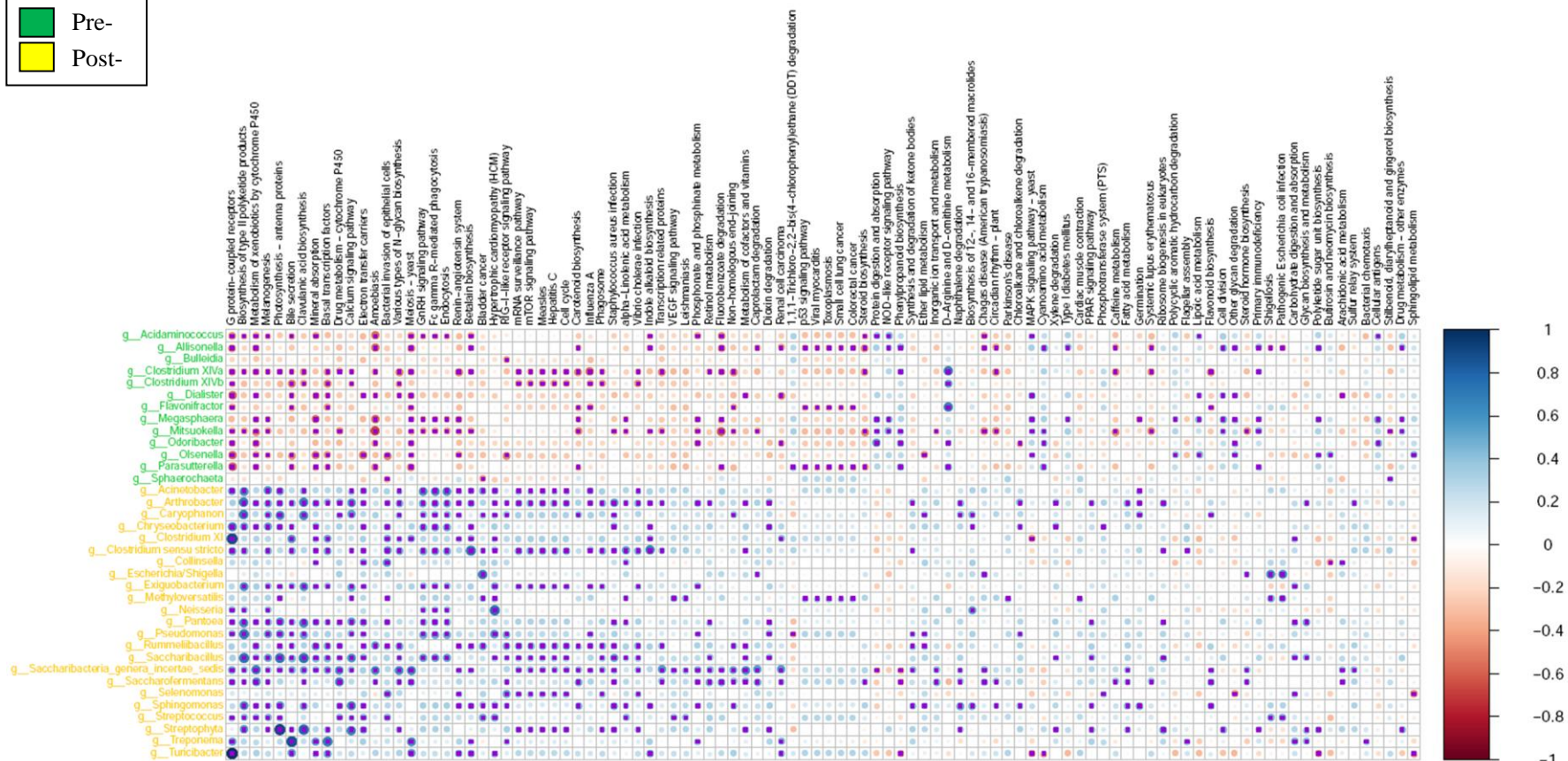

B

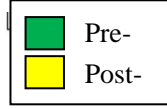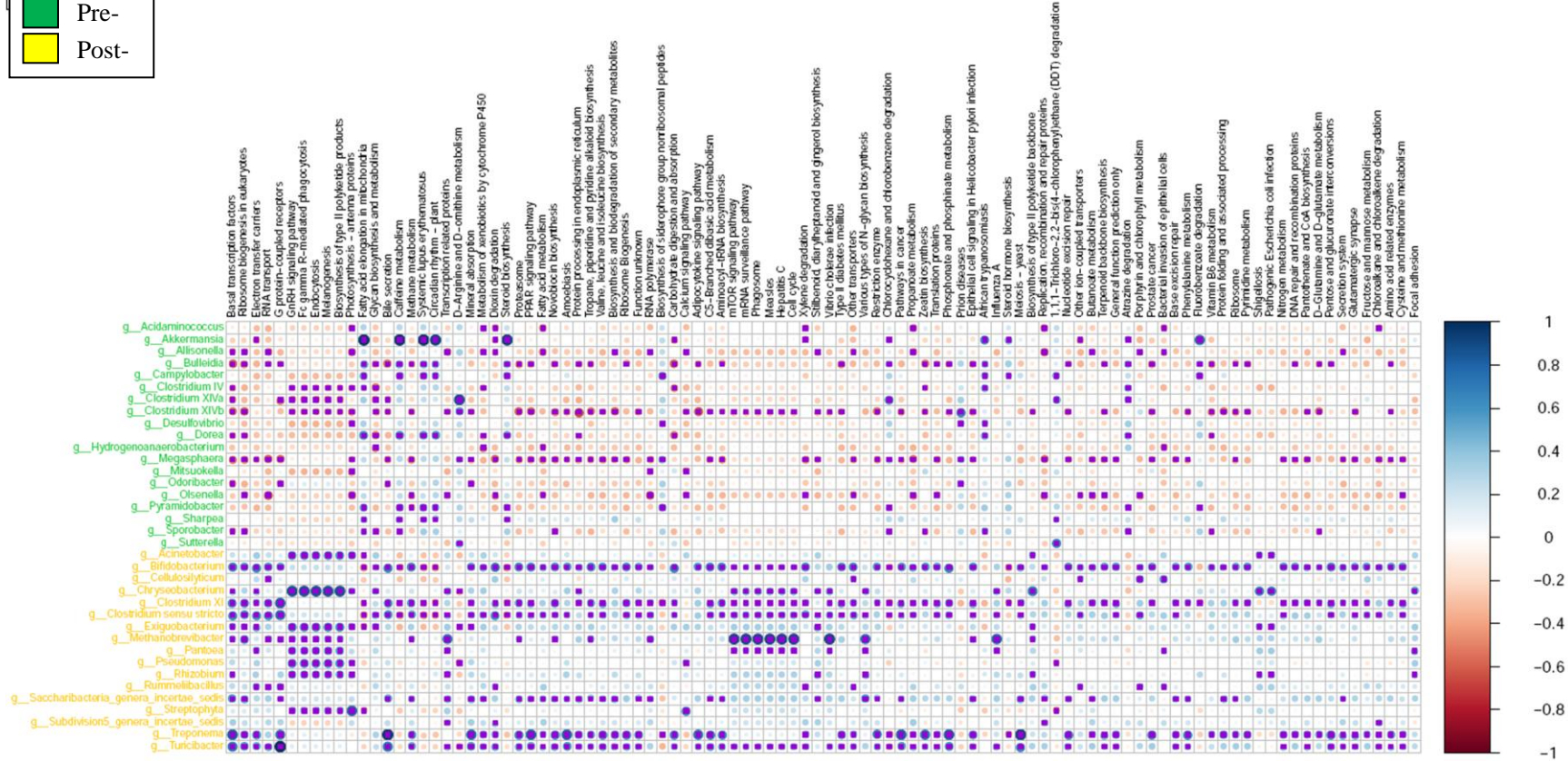

C

Supplement: FIGURE S1 — The age distribution analysis of L (<3.5 months) and H (≥3.5 months) among three intervention groups (A). There is no difference in NG-L, Bs-L, and Ba-L (P = 0.6716), and no difference in NG-H, Bs-H, and Ba-H (P = 0.1073). The PCoA analysis of the OTU difference among three groups after intervention in L (B) and H (C) separately. blue, NG group, red, Bs group, green, Ba group. [file Data_Sheet_1.pdf]
